# From trade-offs to translation: An interdisciplinary roadmap for neurotechnology

**DOI:** 10.1126/sciadv.aee8595

**Published:** 2026-07-31

**Authors:** Ruben Ruiz-Mateos Serrano, Joe G. Troughton, Nima Mirkhani, Natalia Martínez, Massimo Mariello, Jordan Tsigarides, Simon Williamson, Juan Sapriza, Ioana Susnoschi Luca, Antonio Dominguez-Alfaro, Estelle Cuttaz, Nicole Thompson, Sydney Swedick, Latifah Almulla, Amparo Güemes

**Affiliations:** ^1^ Department of Engineering, Electrical Engineering Division, University of Cambridge, Cambridge CB3 0FA, UK.; ^2^ Institute for Biomedical Innovation, University of Cambridge, Cambridge, UK.; ^3^ Institute of Biomedical Engineering, Engineering Science Department, University of Oxford, Oxford OX3 7DQ, UK.; ^4^ Nuffield Department of Clinical Neurosciences, University of Oxford, Oxford OX1 3TH, UK.; ^5^ Department of Electrical and Electronic Engineering, Imperial College London, London SW7 2AZ, UK.; ^6^ Norwich Medical School, University of East Anglia, Norwich, UK.; ^7^ Department of Brain Sciences, Imperial College London, London W12 0BZ, UK.; ^8^ Department of Biomedical Engineering, University of Glasgow, Glasgow, UK.; ^9^ Instituto de Microelectrónica de Sevilla, IMSE-CNM (CSIC, Universidad de Sevilla), Av. Américo Vespucio 28, 41092 Sevilla, Spain.; ^10^ Department of Bioengineering, Imperial College London, London SW7 2AZ, UK.; ^11^ Department of Medical Physics and Biomedical Engineering, University College London, London WC1E 6BT, UK.; ^12^ Department of Clinical Neurosciences, University of Cambridge, Cambridge Biomedical Campus, Cambridge, UK.

## Abstract

Neurotechnologies are transforming how we measure, interpret, and modulate brain-body interactions, integrating real-time sensing, computation, and stimulation to enable precise physiological control. They hold transformative potential across clinical and nonclinical domains, from treating disorders to enhancing cognition and performance. Realizing this potential requires navigating complex, interdisciplinary challenges spanning neuroscience, materials science, engineering, signal processing, and regulatory and ethical frameworks. This Perspective presents a strategic roadmap for neurotechnology development, created by early-career researchers at the intersection of disciplines. We identify five cross-cutting trade-offs that shape the development and translation of neurotechnology: material functionality versus long-term stability, innovative development versus scalable manufacturing, spatiotemporal resolution versus real-time capability, multiscale multimodal integration versus system adaptability, and technological complexity versus clinical or commercial translatability. Rather than a domain-specific review, we focus on shared challenges and strategic opportunities that transcend disciplines, propose a unified framework for collaborative innovation and education, highlight ethical and regulatory priorities, and outline a timeline for overcoming key bottlenecks.

## Teaser

Neurotechnologies face interdisciplinary trade-offs that shape scalable, ethical, and effective translation.

## PREPARING FOR THE EVOLVING LANDSCAPE OF NEUROTECHNOLOGY

Neurotechnologies are reshaping how we understand and influence human physiology, offering tools to record, interpret, and modulate brain-body interactions for therapeutic and augmentative purposes. These systems have already demonstrated the potential to transform health care and well-being by enabling precise, personalized interventions for neurological, inflammatory, and metabolic disorders, cognitive enhancement, and motor rehabilitation. Adaptive and feedback-driven systems (i.e., closed-loop systems) illustrate this potential, seamlessly integrating real-time sensing, computation, and stimulation to modulate neural activity dynamically. By continuously refining stimulation on the basis of physiological feedback, adaptive neurotechnologies surpass traditional open-loop approaches, offering improved therapeutic outcomes with reduced side effects and greater personalization (*1*). This includes a growing emphasis on noninvasive systems, which are increasingly sought after for their accessibility and reduced risk (*1*).

In clinical medicine, closed-loop systems are an established paradigm. Implanted devices [e.g., pacemakers (*2*) and insulin pumps (*3*)] are, in some specialties, standardized and effective means of restoring function to physiological systems. Yet, adaptive systems for disorders of the nervous system are still in their infancy. The neuromodulatory feedback–driven systems closest to widespread use in clinical practice are responsive neurostimulation for epilepsy (*4*, *5*), adaptive deep brain stimulation (DBS) for movement disorders [like Parkinson’s disease and essential tremor and dystonia (*6*, *7*)], and evoked compound action potential–controlled spinal cord stimulation for chronic pain (*8*, *9*). All are iterations on existing open-loop treatments primarily aiming to match the current standard of care while minimizing side effects or the frequency of battery-replacement procedures (*10*). Alongside growing meta-analytic evidence for their use (*11*, *12*), large-scale clinical trials are underway to support broader adoption (*9*, *13*).

Behind these in the translational pathway are open-loop neuromodulation approaches for which closed-loop implementations have yet to be realized. These include peripherally targeted treatments such as dorsal root ganglion stimulation for chronic pain (*14*) and sacral neuromodulation for neurogenic bladder dysfunction (*15*), as well as several forms of noninvasive brain stimulation. Among the latter, transcranial magnetic stimulation is licensed for use in depression, obsessive compulsive disorder (*16*), and migraine, while transcranial direct current stimulation has recently received US Food and Drug Administration (FDA) approval for at-home treatment of depression (*17*). More experimental noninvasive brain stimulation approaches include focused ultrasound, for which a consensus on biophysical safety has recently been reached (*18*), as well as temporal interference stimulation (*19*), which is capable of selectively targeting deep brain regions noninvasively. All are being refined with a view to eventual closed-loop therapy (*20*–*22*). Increasingly, clinical trials of these technologies aim to identify subgroups likely to respond to treatment (*23*), in recognition of the notable heterogeneity among neurological and psychiatric disorders (*24*).

In parallel to the clinical translational pathway, neurotechnologies are expanding beyond therapeutic use. Enhancement of attention (*25*), motor skill acquisition (*26*), learning (*25*), and creativity (*27*) resulting from neuromodulation, particularly in healthy individuals, has prompted suggestions that adaptive systems may be used for a variety of nonclinical applications across several sectors, including education, gaming, athletics, and the military. This expansion has catalyzed rapid industrial growth (*28*). Several companies have focused on direct-to-consumer responsive neurofeedback systems, offering devices for enhanced cognition with ergonomic form factors (*29*–*31*). Others are developer-focused, offering software development kits, application programming interfaces, and hardware for emerging adaptive applications (*32*, *33*). A final group is focused on the development of implantable brain-computer interfaces, leveraging front-end developments in microelectrode arrays and delivery routes (*34*–*37*). This diversification illustrates the field’s fragmentation but also its opportunity space. In 2024, neurotechnology companies received $1.2 billion in venture capital funding (*38*), more than any other type of medical device.

Despite this momentum, the translation of neurotechnologies from research to real-world applications presents challenges. A limited understanding of neural mechanisms in both health and disease constrains identification of effective targets and optimization of stimulation parameters. Consequently, parameters tend to remain within historically determined (and likely suboptimal) norms. Added to this are methodological heterogeneity, small sample sizes, and high interindividual variability, all of which hinder reproducibility and slow discovery. Critically, advancing the field requires integration across multiple disciplines, including neuroscience, materials science, engineering, computer science, and clinical sciences, together with strong clinical and nonclinical translational capacity (*39*), underscoring the need for interdisciplinary collaboration.

Researchers and innovators at all career stages are essential to progress, with early-career researchers (ECRs) being particularly well positioned to drive innovation. Their interdisciplinary training enables them to bridge disciplinary silos and contribute innovative solutions at the interface of physiology and neuroscience, engineering, and translation. However, the complexity of modern neurotechnology systems, from nonadaptive to feedback-driven platforms, emphasizes the need for structured, community-wide efforts to overcome barriers and accelerate progress.

This paper emerges from an interdisciplinary workshop held in Cambridge in November 2024 that convened 20 ECRs from various specializations to examine the current landscape of neurotechnologies and shape its future. We first provide a critical assessment of state-of-the-art limitations within each domain, identifying technological and conceptual barriers that constrain progress in clinical and nonclinical applications. While an exhaustive review of each individual field is beyond the scope of this work, we aim to highlight the overarching landscape and core requirements, with particular emphasis on the cross-cutting trade-offs that constrain progress across adaptive and nonadaptive systems. Here, we use the term “adaptive” to refer to fully closed-loop, feedback-driven systems, “nonadaptive” to refer to open-loop systems without real-time physiological feedback, and “partially adaptive” to describe systems with limited or intermittent feedback. For in-depth discussions, we refer readers to specialized reviews within each area. Subsequently, we highlight key interdisciplinary bottlenecks uncovered through cross-disciplinary discussions, revealing shared trade-offs that transcend individual fields. Drawing from these discussions, we outline strategic directions for advancing the field, emphasizing the need for collaborative frameworks, standardized methodologies and protocols, interdisciplinary education, and ethical considerations in neurotechnology development.

Ultimately, this paper issues a call to action for the broader neurotechnology community, across all career stages, to engage in collective problem-solving and translational research efforts. Only by aligning technical innovation with societal and clinical needs can the field accelerate breakthroughs and shape the future of neurotechnology for both fundamental science and societal impact.

## CURRENT STATE OF THE ART: OVERVIEW, REQUIREMENTS, AND LIMITATIONS

### Material development

**Fig. 1.**
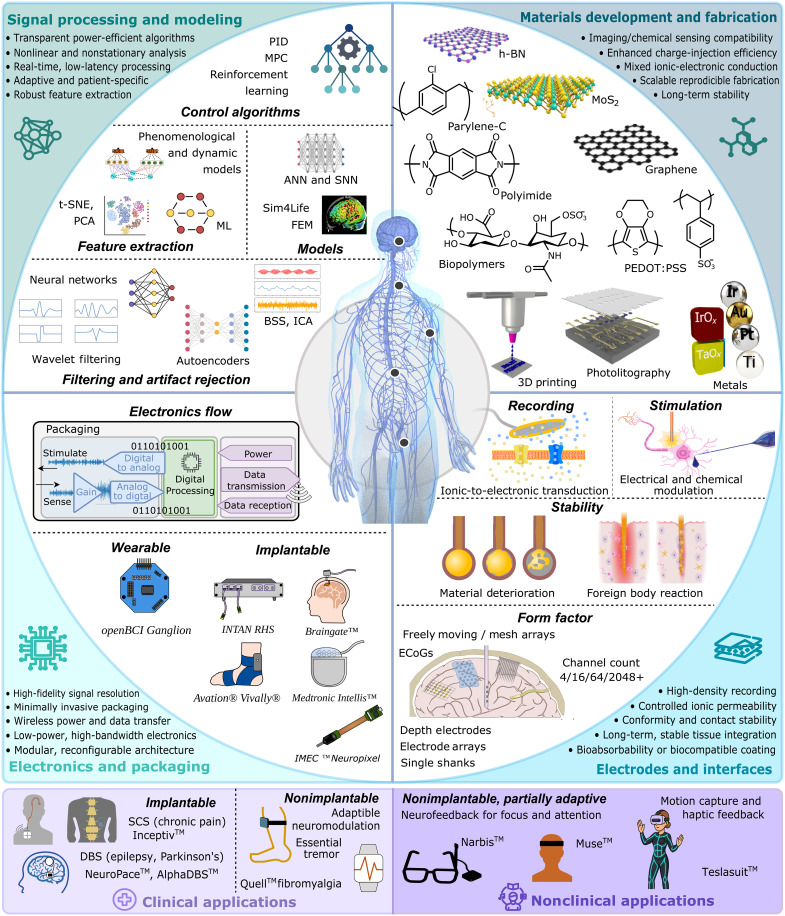
Multidisciplinary landscape and technical requirements for neurotechnologies. This diagram illustrates the current state of the art and critical design considerations across key domains required to advance neurotechnology systems: material development and fabrication, electrode and front-end interfaces, back-end electronics and packaging, and signal processing, with modeling clinical and nonclinical applications shown at the base, emphasizing current translation paths and emerging use cases such as neuroadaptive consumer technologies. The human figure at the middle showcases current locations where these neurotechnologies are being used or to be deployed in the near future. Some elements are adapted from figure 1 in (*40*), licensed under CC BY 4.0. (2021 by the authors, Licensee MDPI, Basel, Switzerland). All other illustrations are original, are created using a combination of Autodesk Inventor and draw.io, and are assembled using Inkscape. All trademarks and brand names are the property of their respective owners and are used for identification purposes only.

Neurotechnologies rely heavily on materials at the tissue-device interface (Fig. 1). While several of these material systems have been extensively reviewed elsewhere (*40*–*44*), here, we focus on the functional requirements and limitations that are most relevant to system-level performance. Traditionally, metals like gold (Au), titanium (Ti), stainless steel, silver (Ag), tantalum (Ta), platinum (Pt) alloys, and cobalt-chromium (Co-Cr) alloys have dominated because of their corrosion resistance and electrochemical properties. These materials have been approved or cleared by the FDA for several neural devices treating conditions such as epilepsy, depression, chronic pain, and pelvic neuropathies (*45*, *46*). However, as devices shrink and demand higher current densities and mechanical compatibility, metals face limitations, including increased impedance and reduced charge injection capacity when miniaturized (*47*). Furthermore, stimulation can lead to toxic by-products because of oxidation-reduction reactions and metal solubilization (*48*).

To overcome these challenges, advanced materials like conducting polymers [e.g., poly(2,3-dihydrothieno-1,4-dioxin)-poly(styrenesulfonate) (PEDOT:PSS) (*49*)] and two-dimensional (2D) materials [e.g., graphene, MoS_2_ (molybdenum disulfide), and h-BN (hexagonal boron nitride) (*50*)] offer improved biocompatibility, electrical performance, and compatibility with nanoscale fabrication (*51*). Yet, technical barriers and failure points remain for many of these materials (*52*), including degradation or loss of functionality when subjected to the rigors of clinical sterilization protocols or long-term implantation (*53*, *54*). Electrochemical stability and mechanical integrity (*52*, *54*, *55*) while maintaining high performance remain a critical challenge, as does enhancing charge injection capacity and minimizing stimulation artifacts (*56*–*59*).

Imaging compatibility is another essential consideration. Materials must not interfere with magnetic resonance imaging (MRI) and computed tomography (CT) scans, which are vital for accurate device placement, diagnostics, and postoperative monitoring (*60*). While neuromodulation paired with imaging enhances precision and feedback (*60*, *61*), traditional metallic implants often cause artifacts, heating, or device malfunction (*62*). This poses risks not only for clinical use but also for consumer-grade neurotechnology, such as wearables that require occasional medical evaluation. Thus, previously unidentified material solutions must prioritize performance, data quality, and safety across diverse applications (*63*–*67*).

### Electrodes and interfaces (front-end)

Closely tied to materials innovation, the design and implementation of electrodes and neural interfaces remain central to neurotechnology system performance (Fig. 1). Yet, limitations persist at the biotic-abiotic interface beyond the materials choice. Early neuromodulation devices relied on labor-intensive manual fabrication methods, which remain prevalent in many commercial systems. While photolithographic techniques have improved throughput and resolution (*61*), their commercial adoption is still limited because of cost and complexity and limits the application of emerging materials.

Electrode form factors have evolved notably since the introduction of the Utah array in 1998 (*62*); however, challenges remain in balancing density, flexibility, and biocompatibility. Increasing the density of recording sites along stiff penetrating electrodes improves resolution but exacerbates mechanical mismatch with neural tissue, leading to chronic inflammation and fibrotic encapsulation. This natural inflammatory and healing response to implants is known as the foreign body response (FBR) and is a major problem that limits the device functionality. Efforts to minimize implantation trauma through shape-changing devices such as expandable devices (*63*–*66*), cortical surface devices (*67*), serpentine and kirigami patterns (*68*–*70*), prestraining (*71*–*73*), and self-wrapping cuffs for peripheral nerves (*74*–*78*) have been promising, but long-term stability and seamless integration remain unproven in humans.

In wearable devices, Ag/AgCl wet electrodes, using conductive gel, are standard for short-term, in-lab recordings because of low skin-electrode impedance and high signal fidelity. However, they require periodic maintenance as gel dehydrates. Dry electrodes, made from materials like metals and textiles, offer a gel-free alternative enabling long-term, ambulatory use, albeit with increased susceptibility to motion artifacts and higher contact impedance. Flexible dry electrodes, incorporating foam substrates or textile-based designs, offer conformity and contact stability, especially over irregular or hairy surfaces such as the scalp. For prolonged monitoring on nonhairy regions, adhesive hydrogel–based electrodes provide stable adhesion and low impedance (*79*).

While cutaneous electrodes provide a simple, safe route for neural interfacing, there is a substantial trade-off with the spatiotemporal fidelity that can be achieved. When an electrode is positioned next to a neuronal membrane, it can record the activity of that single neuron (a single unit). As the electrode size increases and the distance from neural sources grows, the number of neurons’ activity that is being recorded increases. From single-unit activity recorded by small (∼10-μm diameter) intracortical probe electrodes to local and global field potentials captured by the cortical surface and cutaneous electrodes, respectively, the recorded signals reflect progressively greater averaging of neural populations. The required signal acuity therefore dictates the appropriate electrode design (*80*, *81*).

### Electronics and packaging (back-end)

Building on the foundation of materials and interfaces, back-end electronics are the bridge between the neural interface and computational systems, enabling signal acquisition, conditioning, stimulation, and data transmission. Most commercially available neurotechnologies use standard microcontroller-based, printed circuit board–mounted electronics housed in large form factors (*82*), with part or all of the subsystem located externally, either as wearable systems (*83*, *84*) or wired to implanted electrodes (*85*–*87*). Fully implantable systems typically require bulky units mounted in the chest or skull (*88*, *89*), connected to electrodes via long wires. These large form factors are primarily driven by the need to accommodate large batteries for power and data management, as wireless power delivery remains constrained by tissue energy absorption (*90*). This challenge is further amplified by the growing demand for higher channel counts, enhanced spatial resolution, and maintaining the quality of service for high-speed, accurate adaptive systems (*91*). Unfortunately, the leads connected to the electrodes can introduce artifacts and signal degradation, and their integration with bulky designs results in highly invasive devices requiring lengthy surgeries and limiting scalability. Wearable systems, while less invasive and easier to deploy, offer much lower spatial resolution and specificity for recording and stimulating, are unable to access deep neural structures, and interact extensively with the external environment. Hybrid systems combining implanted electrodes and external electronics may achieve high spatial resolution (*92*) but remain vulnerable to environmental factors, infection risks, and limited long-term stability.

### Signal processing and modeling

Neurotechnologies, and in particular closed-loop systems, rely on real-time signal processing and control algorithms to extract actionable information from noisy, dynamic neural data (*93*, *94*), which remains a major challenge (Fig. 1). Traditional denoising methods like wavelet transforms (*95*), independent component analysis (*96*, *97*) and empirical mode decomposition (*98*–*100*) have enabled progress in applications such as seizure detection. However, they often fall short in handling the complexity and nonstationarity of neural signals, especially under real-time constraints. Spatial filtering techniques such as beamforming offer faster artifact rejection (*101*, *102*) but rely heavily on accurate source modeling (*103*). Autoencoder-based models have been proposed to address this gap, yet they still struggle to balance artifact removal with preservation of critical neural signal components (*104*). Emerging hybrid approaches, such as integrating spiking neural networks (SNNs) with wavelets (*105*, *106*), adaptive neural networks (*107*), and state-space models (*108*), offer improved responsiveness but are often too computationally intensive for real-time, multichannel applications.

Feature selection represents a critical aspect in closed-loop neuromodulation. Without a proper mechanistic understanding of the features of neural activity that are causally linked to brain function, even efficient processing algorithms and control strategies risk optimizing for surrogate markers that may not generalize across contexts or individuals. Such a disconnect compromises our ability to shift the targeted behavior or symptom in the intended direction. Automated frameworks, like *catch22*, can extract statistical features (*109*), but clinically relevant biomarkers are still typically found through trial and error, as in DBS (*110*). While machine learning models, including autoencoders and reinforcement learning, show promise in tailoring neurostimulation, they often require extensive validation and are limited by poor generalization across species (*111*). Phenomenological frameworks integrating dynamical systems modeling with machine learning have shown potential in extracting robust biomarkers for real-time neurostimulation (*94*, *112*).

Control algorithms have advanced from basic proportional-integral-derivative (PID) (*113*) to predictive (*114*) and reinforcement learning–based (*115*, *116*) systems, yet real-time deployment in humans remains rare because of computational load in those scenarios. A persistent limitation across modeling and control strategies is poor translatability to human applications mainly caused by interspecies differences in neural dynamics (*117*). Standardized validation protocols, coupled with methods like transfer learning, are therefore essential for improving translatability (*118*, *119*). As the field matures, priority should be given to the development of lightweight, adaptive algorithms that can operate in real time, generalize across patient populations, and integrate seamlessly with sensing and stimulation hardware.

### Ethics and regulatory landscape

The rapid advancement of neurotechnologies offers unbridled potential for treating diseases, from restoring mobility in paralysis to managing chronic pain. However, as UNESCO highlights (*28*), neurotechnologies challenge core aspects of human existence, including mental integrity, dignity, autonomy, and privacy. The very nature of interfacing with the nervous system and the brain, the epicenter of our very being, increases risk of harm and introduces considerable ethical and regulatory challenges. Despite a 268% rise in neurotechnology-related patents between 2013 and 2022 (*120*), an almost 35-fold increase in neuroscience publications between 2000 and 2021 (*28*), and surging government investment worldwide, ethical and regulatory processes remain slow. The evaluation of complex, dynamic systems that comprise materials, electronics, signal processing algorithms, and software poses notable challenges for existing regulatory frameworks, which remain ill-equipped to address these multidisciplinary demands.

Beyond administrative timelines, generating robust safety and efficacy evidence within neurotechnologies is difficult, time-intensive, and costly, particularly for high-risk and implantable systems. This typically requires iterative engineering, preclinical biocompatibility and reliability testing, human factor evaluation, and cybersecurity assurance. Furthermore, adequately powered studies often require long-term follow-up to detect uncommon but serious harms. Consequently, economic analyses of complex therapeutic medical devices (a category that overlaps with many implantable neurotechnologies) estimate mean capitalized development costs on the order of hundreds of millions of dollars once failures and cost of capital are considered (*121*). Where oversight is insufficient, particularly for nonmedical consumer devices, underregulation risks exposing users to avoidable harm, unintended algorithmic behavior, misleading claims, and privacy failures. This reinforces the urgent need for rigorous, risk-proportionate regulation throughout the product life cycle (*122*).

In the UK, the Health Research Authority ethical approvals average 74 days for noncommercial and 113 days for commercial studies (*123*), with subsequent regulatory approvals from agencies such as the Medicines and Healthcare products Regulatory Agency (MHRA) often exceeding a year for class III implantable devices. In the European Union (EU), the Medical Device Regulation (MDR) provides the legal foundation for regulating medical devices including neurotechnologies. In 2022, the MDR expanded its reach to also include certain nonmedical devices including noninvasive brain stimulation systems that may be used for purposes such as gaming or well-being (*124*). This expansion introduced performance-based safety standards and a “maximum acceptable risk” threshold, rather than requiring therapeutic benefit. For clinical neurotechnologies, the requirement of clinical evidence, postmarket surveillance, and notified body assessments leads to delays in approval exceeding 18 months for class III devices. In the US, the FDA regulates neurotechnologies via the premarket approval and de novo pathways. The Breakthrough Devices Program offers an expedited pathway for transformative technologies, but approval for class III devices still averages 12 to 18 months, with investigational device exemptions adding further time before clinical trials.

Data privacy, protected under GDPR (General Data Protection Regulation; EU) and HIPAA (Health Insurance Portability and Accountability Act; US), is critical given the unique sensitivity of neural data. Ultimately, while neurotechnologies promise transformative advances, they face considerable ethical and regulatory barriers. The convergence of prolonged approval timelines, prohibitive development costs, and fragmented international regulations risks stifling innovation and restricting patient access to potentially life-changing interventions.

## CROSS-CUTTING BOTTLENECKS AND TRADE-OFFS IN NEUROTECHNOLOGY

With the landscape and domain-specific limitations now established, it becomes evident that the most formidable barriers to progress in neurotechnology do not exist within these silos but between them: interdisciplinary bottlenecks that reflect competing demands among performance, scalability, usability, and clinical viability. Unlike most medical devices, these systems interact with a highly adaptive nervous system, requiring simultaneous precision in recording and/or actuation and continuous adjustment to patient-specific neural states. This section dissects the fundamental trade-offs that emerge at the intersection of these disciplines, which represent the true grand challenges for the field and outline potential strategies to mitigate or resolve them. Crucially, these trade-offs apply across both adaptive and nonadaptive systems; where the two diverge, this is explicitly noted.

### Trade-off between material functionality and long-term stability

Reliable long-term communication between neural tissue and electronic devices is key for adaptive and nonadaptive neurotechnology. Achieving long-term biocompatibility remains challenging, primarily due to FBR degrading signal quality over time. This challenge is compounded by the extremely small amplitude of neural signals, often in the microvolt range, and the need for devices to interface across delicate barriers such as the blood-brain barrier, which adds further constraints on material choice, size, and invasiveness. To overcome this, future neural interfaces must incorporate materials that reduce impedance and noise while maintaining flexibility and matching the mechanical properties of the surrounding tissue, thereby improving long-term stability and real-time feedback integration without frequent maintenance or replacement. Materials should also exhibit long-term stability against biofluid permeation or corrosion (*125*, *126*) to operate reliably over extended periods. Equally, for wearable brain-computer interface devices, materials must maintain exceptional long-term performance and user experience while ensuring user safety and comfort (*127*–*129*).

Materials with mixed ionic-electronic conductivity have demonstrated high-quality signal transmission from the tissue to device circuits. PEDOT:PSS stands out as a soft and flexible candidate with the capacity to swell in water-based media that present mixed ionic-electronic conductivity. In scenarios where the front-end device is fully polymeric, the water permeability and the mixed condition of PEDOT:PSS enhance neural signal recording (*130*). However, this swelling can also compromise the stability of conventional silicon-based electronic connections at the device’s back-end hardware (electronics and packaging). This problem is further aggravated by micromotion between the implanted devices and the soft neural tissue, causing inflammation, gliosis, and eventual signal loss (*131*, *132*). Addressing these issues requires advanced encapsulation strategies that balance flexibility with hermetic sealing while minimizing adverse immune reactions (*132*, *133*). Beyond fully polymeric implementations, PEDOT:PSS can also be used solely as a coating for metal electrodes, such as plasma-treated gold. Although PEDOT:PSS lacks functional groups for covalent bonding to metals, the silane-based cross-linker (3-glycidyloxypropyl)trimethoxysilane (GOPS) has been demonstrated to react orthogonally with the PSS unit and hydroxyl groups on plasma-treated metal surfaces (*134*) to obtain an improved signal-to-noise ratio (SNR) and ensure good-quality data (*135*–*137*).

On the other hand, materials optimized for neural recording often struggle with charge injection requirements for stimulation, whereas materials engineered for neuromodulation may suffer from high impedance and diminished signal fidelity (*138*, *139*). PEDOT:PSS presents properties that facilitate effective recording and stimulation, two core functionalities in neurotechnology systems (*51*). However, when used for coating metal surfaces, the resulting chemical bond exhibits poor adhesion and instability under hydrolysis conditions, particularly under repeated electrical stimulation (*55*, *140*). Electrical stimulation protocols also affect material longevity, as repeated charge injection can lead to electrode degradation, increased impedance, and signal instability over time (*52*, *139*, *141*, *142*). Optimizing stimulation waveforms, charge balancing, and material composition is crucial in preventing long-term performance deterioration, particularly for chronic implants (*143*). Recently, ambipolar and p-type organic semiconductors have emerged as promising alternatives to improve the efficiency and versatility under recording or stimulation, enabling the transport of electrons as the dominant charge carriers while avoiding issues with regard to mix conduction (*144*, *145*).

Bioresorbable materials constitute another promising pathway, enabling temporary therapeutic solutions without necessitating surgical removal. These materials seamlessly integrate with neural tissues and degrade harmlessly over time. Despite this main advantage, their development and deployment have been limited in clinical contexts, primarily due to difficulties in controlling degradation rates. Biodegradable polymers and bioresorbable metals must maintain mechanical integrity and functional performance for a precise duration before degrading at a predictable rate. Inconsistencies in degradation can lead to premature failure or inflammatory responses. In addition, achieving biocompatibility while ensuring robust electrical conductivity and signal fidelity remains difficult, as many biodegradable materials have inherently poor electrical properties, limiting their effectiveness in recording and stimulating neural activity. Ensuring controlled degradation without sacrificing performance requires meticulous material design (*146*, *147*).

Beyond the development of improved interfacing materials and designs, a further hurdle remains in the connections between these emerging soft, flexible electrodes and the rigid back-end electronics. A number of approaches have been trialed, from traditional methods (e.g., soldering, screen-printing of silver paste, flip-chip bonding, and wire bonding), which are more established especially in industrial settings, to more experimental or nonconventional techniques (e.g., liquid metal bonding, cold welding, conductive elastomers, metal nanoparticle sintering, and soft lithography-based molding) (*148*). Further progress in neurotechnology development will likely depend on interconnection technologies that support high-density electrical routing while minimizing cross-talk and maintaining stability under mechanical and electrochemical fatigue. Such approaches must also mitigate moisture ingress and be compatible with the expanding range of thin, flexible, and stretchable substrate materials (*133*). Establishing robust, scalable, and materials-compatible interconnect architectures therefore represents a critical area for continued research and standardization.

In parallel, efforts toward miniaturizing neural interfaces are shifting these devices from printed circuit board–based systems to wireless millimeter-scale application-specific integrated circuits, system on chips, and system-in-package architectures that can leverage compact, hybrid, flexible, or even wafer-level packaging, reducing, although not eliminating, the challenge of connecting soft and rigid components (*149*). These advances enable minimally invasive implantation, improve biocompatibility by reducing FBR, and enhance long-term stability. These developments increasingly integrate diverse materials and functionalities, such as embedded sensing and on-node processing, into a unified platform, advancing the long-term performance and versatility of closed-loop systems across both central and peripheral applications.

Resolving this trade-off requires a paradigm shift toward co-design, where material properties and long-term stability are considered inseparable from the initial stages of device engineering. This challenge is common to all neurotechnology systems, regardless of whether they operate in open- or closed-loop configurations.

### Trade-off between innovative development and scalable manufacturing

Transitioning novel technologies into clinical practice is a long journey as the innovative materials and device form factors being developed often rely on emerging fabrication technologies that are not yet widely accessible for scalable manufacturing. Now, few facilities are available to scale up device production to a level where comprehensive preclinical testing can be carried out.

Aside from commercially available formulations like PEDOT:PSS, achieving scalability and reproducibility with emerging materials remains substantially challenging. Unlike the well-established silicon-electronics manufacturing, which is based on iterative materials deposition and photolithography steps to build complex, multicomponent electrical interfaces, the development of advanced materials, such as 2D materials (*150*), n-type conjugated polymers, and bioresorbable substrates, requires specialized, lengthy, and synthetic pathways coupled with precise manufacturing to control thickness and uniformity, all of which directly influence device performance (*151*, *152*). However, existing microfabrication and printing techniques are not yet optimized for the seamless integration of heterogeneous materials, miniaturized geometries, and intricate form factors. The complexity of integrating bioresorbable materials with wireless power sources and adaptive systems further intensifies these manufacturing challenges.

Meeting the diverse performance demands of neural interfaces often requires combining multiple specialized materials, which adds fabrication complexity and introduces more potential points of failure. Although recent advances have benefited from silicon-based manufacturing methods, these approaches limit design flexibility and form factor. Moreover, many emerging materials for neural applications are incompatible with conventional semiconductor processes, posing additional integration challenges. Recent trends have shifted toward innovative manufacturing methods that bypass traditional dry and wet etching steps, allowing simultaneous integration of biopolymers and hydrogel-like structures with miniaturized form factors. These increasingly popular techniques for researchers include printed electronics methods such as inkjet (*153*) and screen printing (*49*), conventional extrusion-based 3D printing (*154*–*156*), two-photon polymerization, laser machining (*74*, *157*–*160*), and even in situ fabrication of device components. However, these tools often remain confined to specialized, expensive in-house applications. Integration with microelectronics must additionally meet requirements related to power consumption or heat generation, which are related to tuned material thicknesses, adjustable designs and form factor, and the assembly of conductive and dielectric materials (*161*).

Although individual research institutions can readily achieve small-scale demonstrations of novel devices, wider preclinical investigation and validation require dedicated small-volume manufacturing facilities to transition effectively from academic research to commercial products. While there are a number of contract manufacturing companies that may fulfill this role, accessing these can be challenging. Establishing dedicated scale-up facilities for device production presents a preferable alternative to contract manufacturing. The Center for Process Innovation is a UK-based example of such a facility, aimed at a wide range of technologies including flexible electronics and health care technologies. While the Center for Process Innovation can act as a model in supporting the scale-up of some technologies, their capacity and tooling are limited.

Beyond scale-up requirements, an even greater shortfall exists in manufacturing capacity for clinical trial–ready devices. While a number of global MedTech companies have the capacity in-house for the production of class II and III active medical devices, primarily for control and processing electronics, this remains inaccessible for most development purposes. To promote global development of new devices, tooling must be made available to a wide range of academic and commercial users, rather than being isolated within individual research groups. For wider preclinical trials, platforms must be provided for scaling up both materials and device production and sharing knowledge of these pipelines. Furthermore, complementary facilities must be developed, equipped with the necessary tooling and regulatory compliance frameworks, to produce trial-ready medical devices.

Economically, synthesis and development of innovative materials, implementation of innovative device designs, and miniaturization of neural interfaces require substantial funding and a robust economic framework. Clinical-grade facilities adhering to stringent quality control standards such as Good Manufacturing Practices and Quality Management System are necessary for testing and human trials, even for conventional materials like platinum. In addition, the back-end electronics of neural interfaces necessitate advanced application-specific integrated circuits with high technology readiness levels, as well as powerful hardware to process acquired data. These factors collectively slow the translation of neuromodulation devices.

While these challenges are known to be costly, there is a lack of detailed analyses regarding the funding required to advance ideas from concept to clinical translation. Financial constraints affect not only the testing, validation, and translation of new technologies but also the scalability and integration of these devices into real-world applications. To date, only one study has analyzed the cost-effectiveness of closed-loop spinal cord stimulation compared to open-loop systems, concluding that adaptive systems provide more quality-adjusted life years at a lower cost (*162*). Expanding such analyses to other neuromodulation applications could provide critical insights, fostering collaborative funding strategies, public-private partnerships, and efficient resource allocation. These efforts could accelerate innovation while ensuring economic feasibility. Moreover, demonstrating cost-effectiveness during clinical use could further motivate adoption and highlight the economic advantages of closed-loop systems over conventional open-loop alternatives.

Resolving this trade-off demands coordinated investment in accessible fabrication infrastructure and scalable design pipelines that bridge academic innovation with clinical-grade production. These apply equally across adaptive and nonadaptive systems, as both require clinical-grade production pipelines.

### Trade-off between spatiotemporal resolution and real-time capabilities

The effectiveness of neuromodulation, and particularly closed-loop neuromodulation, depends on how closely recorded signals reflect the underlying neural activity and how precisely stimulation is delivered to target neural circuits. Efficient acquisition, processing, transmission, and actuation of increasing amounts of data are therefore critical. Adaptive neuromodulation systems must therefore balance power, size and latency requirements to achieve clinical uptake.

Increasing the number of electrodes enhances recording and stimulation precision but comes at a cost. Higher channel counts demand complex amplifying electronics, escalating power consumption and device footprint (*163*–*165*). Multiplexing techniques reduce back-end complexity but degrade signal quality in the time or frequency domains (*166*–*168*). This trade-off between fidelity, cost, and power efficiency remains a major hurdle for real-time, high-density neural interfacing.

Ideal electrodes accurately capture neural signals while minimizing external interference. A high dynamic range allows the system to detect weak neural signals and strong transient artifacts without losing critical information. This requires advanced signal processing techniques where amplification and filtering can also introduce additional noise, ultimately degrading signal quality.

Adaptive channel selection is another key enabler, continuously activating the most informative channels while suppressing noisy or redundant data, enhancing spatial resolution. This is particularly beneficial with the proliferation in high-density electrode arrays, necessitating the extraction and processing of large quantities of neural activity. Nevertheless, the quality of the acquired data still heavily relies on electrode design and placement. Poor electrode placement or design can lead to high impedance and noise, reducing recording quality.

Data throughput can be enhanced by increasing on-chip processing, but this comes at the cost of higher power requirements. Increasing the available power, however, remains a challenge, particularly in wireless systems. Electromagnetic power transmission is the most mature method but is limited by battery life, restricted range for temperature increases, and antenna footprint as well as by tissue absorption (in implantable devices). Proper resource management is thus crucial to enable portable, power-efficient devices that are more likely to be adopted in clinical practice. Alternative power and data transfer strategies have been proposed including high-frequency ultrasound and near-infrared, although each carries inherent trade-offs related to biocompatibility and integration complexity. Advances in predictive modulation and wireless data transmission also help to minimize data transmission requirements, conserving both energy and bandwidth.

The data requirements for closed-loop neuromodulation are often vast, exceeding the capacity of current processing architectures, particularly the need for real-time processing and feedback. While deep learning and high-dimensional signal processing approaches provide the necessary performance, their power requirements remain well beyond what is available in stand-alone devices. Current research is exploring a variety of innovative solutions to this. Hardware acceleration, particularly through graphics processing units (GPUs) and field-programmable gate arrays (FPGAs), can provide the necessary computational power, while algorithmic optimization ensures that only the most relevant data are processed. Hybrid approaches, which dynamically adjust model complexity, offer the flexibility to balance performance and efficiency. In addition, compression methods such as adaptive sampling and time-division multiplexing allow systems to reduce the amount of data they handle, substantially lowering power consumption. Meanwhile, machine learning techniques focused on feature extraction offer the potential to automatically identify and prioritize important data features for more efficient processing.

Last, neuromorphic computing, inspired by the brain’s architecture, is emerging as a promising avenue to reduce power requirements for on-chip processing, offering a route to energy-efficient, low-latency processing capabilities for both signal detection and neurostimulation (*169*).

Resolving this trade-off requires a multipronged approach combining optimized electrode design, adaptive signal processing, and energy-efficient architectures, such as neuromorphic computing, to balance resolution, latency, and power consumption in neurotechnology systems. While these constraints affect all neurotechnology systems, they are particularly acute in adaptive closed-loop systems, where real-time processing and low latency are essential for effective feedback control.

### Trade-off between multiscale multimodal integration and adaptability

Advancing neurotechnologies requires greater adaptability across physical and software domains to accommodate a wide range of deployment scenarios, during both data acquisition and stimulation, while accounting for intra- and interuser variability. Adaptability in neurotechnologies is also uniquely challenging because the brain and user are themselves adaptive across many timescales, from rapid synaptic plasticity and state-dependent modulation to long-term learning and behavioral changes. Devices therefore face a moving target, where performance must be maintained despite ongoing neural and behavioral adaptation. This inherent adaptability of the nervous system directly conflicts with scalability: With limited training data, systems are unlikely to generalize well across diverse users or scenarios, and accounting for such variability requires large and heterogeneous datasets. Effective platforms must therefore dynamically adjust to diverse temporal resolution and spatial coverage demands across multiple modalities, enabling precise targeting, accurate event detection, and personalized responses. Adaptability also extends to applications like brain-computer interfaces, well-being monitoring, and gaming. To ensure generalizability, systems must perform reliably across populations with diverse physiological traits (e.g., hair type, skin conductance, age, and gender) while balancing wearability, form factor, cost, and data fidelity.

However, developing more adaptive systems faces a deadlock: High-quality results typically require extensive data acquisition from hundreds of channels, which conflicts with size and power constraints. A way forward is through distributed systems, where minimally invasive, flexible, and miniaturized sensors collect data close to the zone of interest while conforming to the body’s shape and movement (*92*). These can support multimodal data acquisition and adaptable stimulation, enabled by low-heat dissipation electronics and wireless power and data transfer. Processing is increasingly off-loaded to the edge: Local feature extraction, channel selection occurring near the sensor (on-the-fly processing), and event-based sampling minimize transmission and power. Although current signal processing techniques are not fully compatible with event-driven methods, asynchronous approaches like spike-based neural networks and event-driven systems (*170*) can bridge this gap.

More powerful computational platforms can be placed in less-constrained locations (e.g., chest units or wearable devices), ensuring low-latency inference, improved energy efficiency, and data privacy. Central processing unit–based platforms are suited for evolving inference algorithms without hardware translation overhead, while reconfigurable circuits and neuromorphic computing enable real-time processing through parallelization, data quantization, and memory-efficient designs [e.g., static random-access memory (SRAM) over dynamic random-access memory (DRAM)]. Fully neuromorphic pipelines, from feature extraction to decision-making, allow for low-latency and energy-efficient operation while preserving algorithmic flexibility.

While recent advances in neuroprosthetic systems, such as speech decoders (*171*), demonstrate substantial capabilities, most traditional approaches that rely solely on electrical recordings still face limitations in capturing the rich biochemical and physiological context underlying neural function. Integration of optical and biochemical sensing provides real-time monitoring of metabolic, hemodynamic, neurotransmitter, and inflammatory markers for a greater understanding of the physiological environment and signal dynamics, which are increasingly recognized as important for understanding the physiological environment and supporting the long-term stability of adaptive implants. Combining modalities (e.g., electrical, optical, and chemical) enables more comprehensive interaction with neural systems, allowing both sensing and stimulation to be tailored to the specific physiological phenomenon of interest. Different approaches bring distinct strengths and limitations. For example, imaging techniques (e.g., functional MRI and calcium imaging) provide high spatial resolution but slower dynamics, while chemical sensing (e.g., neurotransmitters and pH) yields rich contextual data but may be affected by signal drift or lag. Rather than requiring every system to achieve all functions, the choice of modalities depends on the target process and intended therapeutic or scientific application. To overcome these issues, a promising approach involves combining phenomenological models of delay-coupled nonlinear systems with forward models (e.g., Balloon Windkessel). These frameworks account for dynamic interactions and time delays, enabling a comparison of distinct features that are captured across multiple modalities.

Materials choice also critically influences multimodal integration, as many conventional electrode metals produce imaging artifacts. Promising electrode materials, such as carbon materials (e.g., graphene and carbon nanotubes) (*172*) as well as conducting polymers (*173*), have shown strongly reduced artifacts for both MRI and CT. Alternatively, optical fibers, replacing wires for photonic power transfer, have demonstrated full MRI safety (*174*). Integrating multifunctional materials for multimodal sensing, stimulation, and drug delivery is also a promising avenue. Materials such as magnetic nanoparticles for magnetothermal neuromodulation and plasmonic nanoparticles for optogenetics allow real-time biochemical monitoring optimized via machine learning (*175*). While hybrid designs incorporating bioresorbable, ionic-electronic, or optically active components hold great promise, they require innovative encapsulation techniques, often compromising application-specific performance.

Given patient response variability, identifying structural and functional neuroimaging biomarkers is key for adaptive neuromodulation. Hybrid models, combining finite element analysis with neural networks, offer promising solutions for integrating real-time stimulation dynamics with adaptive responses, addressing scalability and computational challenges (*176*). These techniques improve the modeling of nonlinear interactions across networks and capture multiscale dynamics, advancing adaptive applications while maintaining the physiological accuracy required for clinical and nonclinical implementations (*177*). Cloud platforms may support the aggregation of multiscale data by combining macroscopic connectome data with neural population models across patients, but this approach must be carefully balanced against concerns around data privacy, autonomy, and system dependency. Rather than relying on continuous internet connectivity, we envision secure, locally hosted or edge-computing solutions that enable patient-specific calibration and algorithm training without compromising user agency or safety. Achieving effective multimodality generalization and personalization requires large, standardized datasets and proper testing frameworks. Nonclinical applications have the power of providing data from a wider range of scenarios for this cause, enabling more streamlined personalization to future users.

Resolving this trade-off will require distributed, event-driven architectures, multimodal sensing platforms, and hybrid modeling frameworks that together enable scalable personalization and robust adaptability across diverse physiological and deployment contexts. While multimodal integration is relevant across all neurotechnology systems, the adaptability dimension of this trade-off is particularly pronounced in closed-loop implementations, where systems must dynamically respond to continuously evolving physiological states.

### Trade-off between technological complexity and clinical or commercial translatability

In general, the development of the next generation of neurotechnology systems follows two pathways: developing novel neurotechnologies from in silico/in vivo preclinical models or upgrading existing open-loop solutions to incorporate closed-loop capabilities. In either case, the effective translation of neurotechnology systems for clinical and commercial use depends fundamentally on high-quality evidence, ethical and regulatory approval (*178*–*180*), and stakeholder acceptance. The relevant stakeholders include patients, clinicians, regulatory bodies, health care providers, researchers, and commercial end-users such as gamers. Each group plays a crucial role in the adoption and integration of these technologies into practice. Ensuring acceptance and usability requires careful consideration at all levels of development, from material selection and device design to data interpretability and user interfacing and comfort. Technological complexity at any level can be a barrier to translation.

At the level of materials, substantial complexity arises from interactions between device components and biological tissue, particularly for implanted devices, which carry the risk of an adverse tissue response; conventional rigid electronics can also restrict mobility and cause discomfort. Even minor differences in material composition can lead to inconsistencies in device performance, complicating cross-study comparisons and hampering the development of standardized manufacturing processes. The greater the complexity of these interactions and their downstream effects on device function, the greater the likelihood of additional regulatory bottlenecks and limited widespread adoption (*178*–*180*). Material design must also be optimized for efficient and safe surgical handling during implantation procedures, which, in turn, must minimize the risk of tissue damage and be simple enough to be performed routinely. The diversity of form factors requiring bespoke procedures will result in discrepancies in clinical outcomes (*181*).

Once implanted, maintenance of the device is an important concern. Many current neurotechnology systems require regular recharging or invasive battery replacement. This in turn increases patient burden, limits accessibility, and raises ethical questions regarding equitable care distribution (*179*, *182*, *183*). Furthermore, device failure, such as through biofouling or material degradation, not only compromises function but also requires additional invasive procedures to remove or replace the device, introducing further clinical and ethical complexities (*179*, *184*). Without ongoing support, these devices risk becoming “abandonware” (i.e., remaining in the body without updates, maintenance, or serviceability), posing substantial threats to patient safety (*182*, *183*). User-driven abandonment is also a concern: Devices considered intolerable may be disengaged before meaningful long-term data regarding their effectiveness can be gathered (*185*, *186*). To prevent this, patients should be informed of the uncertainty regarding the long-term risks of implanted devices before providing consent, and manufacturers must ensure compliance with ISO standards as a benchmark for safety and reliability to comply with stringent regulatory frameworks (*180*).

Regarding data and power transmission, wireless technology has well-established protocols from Internet of Things (IoT) with several explored modalities (WiFi, Bluetooth Low Energy, radio, etc.); however, a balanced solution between wireless power transfer and wide data rate remains underdeveloped across both clinical and commercial settings (*180*). Existing regulatory approval frameworks often struggle to accommodate emerging power technologies, delaying the adoption of innovative energy solutions (*178*, *179*) and causing disparities in access to safe and sustainable power sources (*124*, *182*, *184*).

The signal processing and adaptive algorithms necessary for closed-loop systems are additional sources of technological complexity. A direct strategy is upgrading open-loop systems by leveraging existing clinical and technical infrastructure, allowing developers to incrementally model and refine the feedback and control elements specific to adaptive operation. However, this path presents unique challenges. Retrofitting closed-loop functionality into existing platforms may be constrained by hardware limitations, lack of integrated sensing, or insufficient understanding of the appropriate biomarkers and control algorithms. As a result, upgrades often require major redesigns to support real-time data acquisition, processing, and actuation while maintaining clinical usability and regulatory compliance.

On the other hand, for novel technologies, a major barrier is accurately modeling healthy or diseased neurophysiology. This is essential to effectively translate preclinical strategies but challenging as neural processes are predominantly nonlinear (*187*) and anatomical variations can limit direct translation of devices and stimulation dosages (*188*, *189*). Artificial intelligence (AI)–enhanced personalized imaging may enable detailed mapping of the brain and peripheral nerves, which is likely crucial for individual-tailored adaptive systems. With fully mapped and characterized neural substrates, it becomes possible to implement real-time feedback systems for accurate sensing, monitoring, and adaptive stimulation control. However, complex models and their associated systems present ethical and regulatory concerns regarding data privacy, algorithmic transparency, and generalizability (*124*, *182*, *183*). Regulatory requirements prioritize interpretability, and clinical workflows require it, which restricts the use of black-box models unless their decision-making can be explained (*178*, *182*). Mandates on validation, data privacy, and security ensure reliability but potentially slow innovation (*180*, *183*). Regarding the use of AI for closed-loop control, traditional approval pathways struggle with real-time adaptation, as regulations are designed for fixed algorithms rather than dynamic models. While recent frameworks, including the EU AI Act and MHRA’s AI Airlock initiative, aim to introduce adaptive oversight mechanisms, striking a balance between innovation and accountability remains a key bottleneck in AI-driven neurotechnologies (*190*).

A final barrier to translation is the heterogeneity that pervades much of the preclinical, clinical, and nonclinical research into neuromodulation. Variability in study protocols and methodologies heavily influences how neuromodulation devices are designed, packaged, and implemented. In clinical trials, methodological variability paired with small sample sizes severely limits the external validity of findings and, thus, the potential for generating meta-analytic evidence necessary for clinical uptake. In addition, inaccuracies in clinical diagnosis, undetected comorbidities, and the inherent heterogeneity among clinical populations can distort both the selection of biomarkers and the corresponding stimulation paradigms. This weakens the causal link between symptom severity and modulation outcomes (*191*). Compounding this, the reliance on historically determined stimulation parameters rather than the mechanistic understanding of how to engage specific neural networks risks limiting the efficacy of even technically advanced systems. Developing principled, network-informed frameworks for parameter selection therefore represents a critical unmet need across both adaptive and nonadaptive systems.

Small studies with divergent designs also yield inconsistent data formats and variable data quality, complicating the creation of robust, generalizable machine learning algorithms (*192*). Models often become overfitted when trained on limited or highly variable datasets, hindering their ability to perform across broader populations. The extensive parameter space in closed-loop systems, ranging from different neural biomarkers to a multitude of stimulation parameters, makes it difficult to compare or replicate findings across studies (*193*–*196*). To overcome these issues, researchers must develop standardized datasets that reflect diverse patient traits and conditions (*197*, *198*). Validating these approaches requires rigorous effect size evaluations, adequate sample sizes, and clear clinical relevance, which are benchmarks that can be difficult to achieve without consistent methodologies and regulatory alignment.

In summary, even the most advanced neuromodulation technology will face adoption barriers if it does not align with regulatory requirements or the needs of stakeholders. Both of these, in addition to large, robust, and methodologically consistent human trials, are necessary for the deployment of neurotechnology systems for clinical and nonclinical applications. Engaging stakeholders throughout development, incorporating feedback early, and refining verification and validation processes postrelease are crucial for ensuring successful implementation and long-term usability.

Resolving this trade-off, developers must embed usability, interpretability, and stakeholder engagement into every stage of design, ensuring alignment with regulatory and translation workflows. Translational barriers affect adaptive and nonadaptive systems alike, although closed-loop systems face additional complexity in regulatory approval because of their dynamic, algorithm-driven nature.

## INSIGHTS GATHERED FROM DISCUSSION OF TRADE-OFFS

**Fig. 2.**
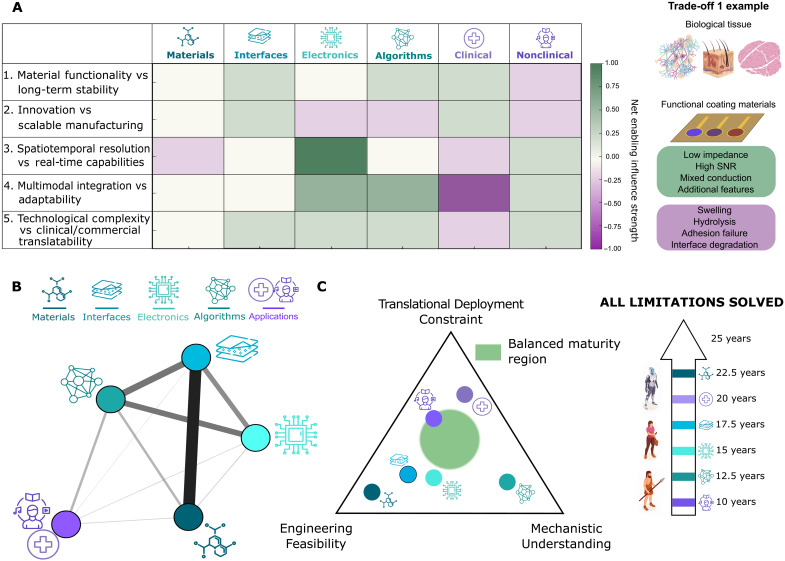
Multidimensional analysis of technical trade-offs and collaborative strategies in neurotechnology development. (**A**) Influence matrix showing how key technical domains (Materials, Interfaces, Electronics, and Algorithms) and application domains interact across five major trade-offs in neurotechnology development (1, material functionality versus long-term stability; 2, innovation versus scalable manufacturing; 3, spatiotemporal resolution versus real-time capabilities; 4, multimodal integration versus adaptability; 5, technological complexity versus clinical or commercial translatability). The color intensity represents the net strength of influence (green: enabling; purple: constraining) between domains for each trade-off. A representative visual example of trade-off 1 is also illustrated. (**B**) Connectivity matrix depicting interactions between domains involved in addressing the trade-offs. Nodes represent domains, and connecting lines indicate cross-domain interactions, with the line thickness reflecting the relative importance of each interaction. (**C**) Conceptual framework positioning the domains within a triangular space defined by Engineering Feasibility, Mechanistic Understanding, and Translational Deployment Constraints. The central green circle represents the balanced maturity region that all domains must converge toward. The accompanying timeline illustrates the perceived time horizon for a new generation of neurotechnologies capable of resolving the identified trade-offs across each domain on the basis of consensus discussions during and after the ECR workshop. All graphical elements are original and assembled using Inkscape.

Understanding the role of each technical domain in shaping key trade-offs is crucial for advancing open-loop and closed-loop neurotechnologies. Figure 2A presents an influence matrix developed by ECRs that maps how each domain can either facilitate or constrain progress, helping to identify targeted strategies to improve both reliability and efficiency. To generate the matrix, each author provided short bullet-point lists of enablers and blockers for every trade-off-domain cell. No weighting was applied at the input stage. Enabler (*E*) and blocker (*B*) scores were then computed by simple frequency counting of these bullet points across authors for each cell. The net influence score was defined as $I_{\text{net}}=\frac{E-B}{S_{\text{max}}}$, where ($S_{\text{max}}=5$). Values were bounded to [−1, 1], where +1 indicates fully enabling consensus, −1 indicates fully blocking consensus, and 0 indicates a balanced or neutral influence. These values were then mapped to a color code to facilitate interpretation. As a result, green indicates that a domain acts predominantly as an enabler, purple indicates that it acts predominantly as a constrainer, and white indicates that its net influence is balanced. Interfaces emerge as a key enabler for trade-offs 1 and 2, reflecting their central role in bridging material innovation with functional device integration. Electronics acts as an enabler across trade-offs 3, 4, and 5, consistent with the rapid advances in processing, acquisition, and adaptive hardware. Algorithms similarly enables trade-offs 4 and 5, reflecting the growing maturity of computational and algorithmic tools for multimodal integration and translational workflows. In contrast, Materials constrains trade-off 3, highlighting the unresolved challenges in achieving the spatiotemporal performance required for real-time applications. Electronics and Algorithms both constrain trade-off 2, reflecting the difficulty of scaling innovative hardware and computational models into reproducible manufacturing pipelines. On the other hand, Interfaces constrains trade-off 5, underscoring the complexity of translating advanced interface designs into clinically viable and regulatory-compliant systems. Last, clinical and nonclinical applications sit above the technical matrix as overarching constraints, shaping priorities and defining success metrics for the development process as a whole.

While Fig. 2A provides a snapshot of which domains support or hinder progress, it does not capture the complexity of multidisciplinary collaboration needed to resolve trade-offs. Figure 2B addresses this gap by depicting domain interactions as a connectivity network, where each node is color coded by domain and line thickness reflects the importance of each relationship, and by identifying the specific collaborative opportunities these interactions represent. The strongest interaction exists between Materials and Interfaces, reflecting their tightly coupled roles: For trade-off 1, achieving both material functionality and long-term stability requires tight coordination between these domains, as material scientists must understand available interfaces and front-end fabrication processes to design compatible materials, while manufacturing engineers must adapt their designs to accommodate emerging material properties. Similarly, the advancement of innovative development and scalable manufacturing (trade-off 2) relies heavily on Interfaces while still requiring critical input from Materials. The relationships between Algorithms and Interfaces, and between Algorithms and Electronics, are also prominent: Addressing trade-off 3 necessitates coordinated efforts across Interfaces, Electronics, and Algorithms, where each domain must compensate for the others’ limitations, while trade-off 4 primarily involves Interfaces and Algorithms, with signal processing engineers developing methodologies that streamline integration tasks, together with optimized hardware to deliver more robust data for downstream analysis. Clinical and nonclinical applications are each linked to the network through Algorithms, reflecting that trade-off 5 (the translation of technological complexity into clinical or commercial viability) requires close interaction between end users and software engineers, with user-facing components that are well documented and accessible, and materials that are safe, reliable, and durable.

Figure 2C positions each domain within a triangular space defined by Engineering Feasibility, Mechanistic Understanding, and Translational Deployment Constraints, revealing the current maturity landscape across the field. Materials, Interfaces, and Electronics cluster toward the Engineering Feasibility vertex, indicating that progress in these domains is primarily driven by technical and fabrication advances. Algorithms sits closest to the Mechanistic Understanding vertex, reflecting its dependence on deeper knowledge of neural function to reach its full potential. All domains remain distant from the central green circle that marks the balanced maturity region, although Electronics and Interfaces are relatively closer, suggesting that they are furthest along the path to balanced maturity. Clinical and nonclinical applications both sit toward the Translational Deployment vertex, with nonclinical applications already touching the balanced maturity region and clinical applications in close proximity, reflecting the more immediate readiness of consumer and nonmedical contexts relative to regulated clinical use. The accompanying timeline illustrates the expected horizon for resolving each fundamental trade-off, as estimated by ECRs across all domains. The longest estimated timeline is associated with Materials, projected at ∼20 to 25 years, with subsequent topics reducing their estimated time by ∼5 years. Timelines vary notably between clinical (20 years) and nonclinical (10 years) application contexts. This disparity is consistent with the triangle positioning: Nonclinical applications have already reached the balanced maturity region, suggesting that they are poised for deployment once the underlying technical trade-offs are resolved, while clinical applications must proactively address the additional translational and regulatory demands that currently keep them from the same threshold. Strategies should be considered to accelerate the readiness of the clinical use space, ensuring preparation for adoption once the underlying technologies are mature.

## FUTURE OF NEUROTECHNOLOGIES

**Fig. 3.**
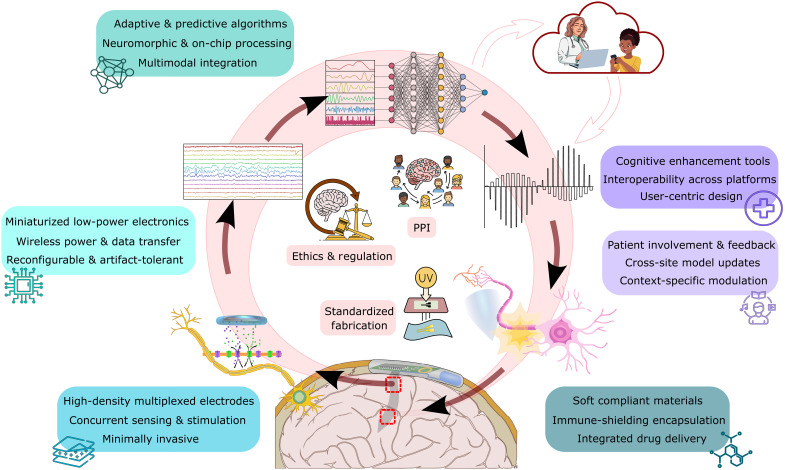
Vision of the future for closed-loop neuromodulation. This schematic depicts the journey of neural signals from detection of single neuron depolarization events (spikes), through recording of many-site aggregated signals (local field potentials), and the deconvolution of these into discrete brain waves onto the processing and clinical decision-making and, lastly, the therapeutic neuromodulation intervention. At the core of this progress lie ethical and regulatory considerations, PPI, and the need for standardized fabrication processes. Each step has many possible futures; the text reflects our collective vision of what an adaptive neuromodulation system should comprise. Some graphical elements are adapted from resources designed by Magnific (www.magnific.com) under a free license. All other illustrations are original, are created using Blender, and are assembled using Inkscape.

Closed-loop neurotechnology systems have the potential to revolutionize the clinical treatment of chronic disorders and, in so doing, substantially relieve the burden of global diseases (*199*). However, it is important to note that effective modulation of neural activity does not necessarily guarantee improved function. Interventions may yield no substantive effect or could produce acute or long-term adverse consequences that must be carefully monitored. Our collective vision on the future of adaptive neuromodulation is depicted in Fig. 3. Within the next 5 years, we anticipate transformative progress in correcting localized anatomical deficits where the intended effect is known and the clinical benefit is measurable [e.g., spinal cord bypasses (*87*, *200*), neurally integrated prostheses (*201*), and advanced speech decoding (*202*)]. These advances will be likely enabled by early resolution of trade-offs in electronics, interfaces, and signal analysis. Within the same time frame, effective application of closed-loop systems to brain disorders characterized by altered connectivity and network dynamics (*203*) may gain traction aided by advanced individualized models of the brain (*204*, *205*) as multimodal (*206*) and multisite (*207*) integration and adaptability improve (trade-off 4). Their full scope of application, however, is expected to broaden. Within the next 10 years, it will potentially encompass conditions beyond the nervous system such as cancer (*208*), functional gastrointestinal disorders (*137*, *209*), diabetes (*210*, *211*), and overactive bladder (*212*), as scalable manufacturing and real-time capabilities mature (trade-offs 2 and 3). Powerful models, comparable to today’s large language models (*213*), will likely emerge from vast amounts of anonymized data, uploaded from individual devices to a shared cloud database (*214*). Such foundation model–like approaches can reshape the field at multiple levels. On the signal processing front, they give rise to transferrable learning across disorders, devices, and patient populations. At the control level, they bring about the transition from reactive, biomarker-triggered stimulation toward predictive, state-aware modulation. Together, given the expanding capacity of AI in extracting meaningful patterns from big data and generating predictions for the corresponding systems, we expect a notable boost in closing the epistemic gap between our ability to modulate activities and causal understanding of neural dynamics, thereby supporting clinical decisions at scale (*199*, *205*).

In 5 to 10 years, we can expect the commercial neurotechnology sector to refine data acquisition (*215*–*217*), implantation (*218*), and brain-computer interfacing to a point where communication is seamless; cognitive domains such as decision-making, memory, and attention can be modulated (*219*); and productivity is enhanced across sectors, all driven by progress in usability and nonclinical translatability (trade-off 5). Accordingly, we also expect expansion into sectors such as education [for learning (*220*)], sports [for reaction time and muscular enhancement (*221*)], entertainment [for responsive virtual environments (*222*) and biologically attuned virtual avatars (*223*)], and the military [for enhanced physical and mental capabilities (*224*–*226*)]. At this point, the ubiquitous generation of “neurodata” may well incentivize external parties, such as employers, marketing entities, or governments, to tailor their strategies for management, advertising, or policy to leverage insights from neural data and optimally influence human behavior (*221*). Questions as to the legality of data sharing and access will be of utmost relevance (*227*), necessitating a balanced approach to regulation (*228*).

As systems advance further, we may see the incorporation of an “external loop” designed to monitor the user’s external environment in real time (*229*). This will enable context-specific modulations, such as preemptive calming in the event of posttraumatic stress disorder (PTSD) triggers or a sense of reward when presented with healthy food options, to treat disorder and enhance desirable behavior, respectively. In several decades’ time, after years of hardware and software refinement, once long-term stability and regulatory integration are achieved, precision electronic medicine in the form of neuron-level control may be achieved (*230*). In such a scenario, even the most complex disorders may become tractable, although fundamental limitations will remain. Treatment will be a case of “rehabilitating” aberrant nervous systems to within normative bounds or of altering a neurodevelopmental trajectory to prevent the onset of illness entirely (*231*). AI-driven decision-making will likely play a big role in determining the aforementioned bounds and trajectories. At a societal level, the ubiquitous enablement of superhuman cognition would allow for unprecedented levels of productivity and, arguably, the ushering in of a technological singularity (*232*).

At an even earlier stage, highly innovative methods offering fundamentally less invasive, and non–electrode-based ways to modulate neural activity are in active development, although not yet mature for clinical translation. Unlike conventional electrode-based systems, these approaches exploit physical and biological mechanisms to achieve neural modulation with greater spatial specificity and reduced invasiveness. Examples include photothermal wireless DBS (*233*), which uses near-infrared light to trigger endogenous, heat-sensitive receptors; sono-optogenetics (*234*), which combines the penetration depth of ultrasound with the cell specificity of light without the need for invasive fiber-optic implants; and magnetomechanical techniques (*235*), which use magnetic nanoparticles to induce torque on mechanically sensitive ion channels when induced by an external field.

Although the possibilities are inspiring, a realistic view of the future must be tempered by consideration of the likely limitations. Some conditions may be too complex for even advanced systems to counteract (e.g., genetic syndromes), while others involving irreparable damage (e.g., cerebrovascular accident) are likely to set a hard limit on what neuromodulation can achieve. There is also the risk, as outlined in the above sections, of immediate or long-term damage to the nervous system as a direct consequence of neuromodulation, which the field must remain vigilant toward. Nevertheless, we remain cautiously optimistic that the future of closed-loop neurotechnology will be a net benefit for global health and well-being.

### Preparing for the ethical landscape of tomorrow’s neurotechnology

Neurotechnologies raise urgent ethical and regulatory challenges that must be addressed proactively, not retroactively, as they move from research to real-world use. Ethical frameworks must address issues of ongoing consent, longitudinal feedback effects, and shared liability among clinicians, manufacturers, and developers. In addition, closed-loop systems that autonomously adapt to physiological and behavioral states in real time introduce previously unknown risks beyond traditional neurotechnologies. Their ability to shape neural activity over time raises concerns about loss of user agency, unintended behavioral effects, and long-term neuroplastic consequences. Because adaptive systems dynamically evolve postdeployment, traditional models of one-time informed consent and fixed accountability are insufficient. In addition, users and clinicians must be able to understand, audit, and intervene in system behavior over time.

These require multidisciplinary collaboration between ethicists, regulators, clinicians, engineers, and patient groups. Ethical guidelines should evolve alongside flexible and adaptive regulatory frameworks, establishing globally harmonized standards for algorithmic transparency, data security, privacy, and autonomy. Standardized patient and public involvement (PPI) guidelines should be followed by clinicians and researchers from early design stages to ensure long-term bidirectional trust, equity, and relevance, and minimize the risk of user abandonment resulting from discontinuation of support (*236*). PPI participants should have a say in shaping guidelines regarding the ethical use of these technologies.

Expanding on this idea, development pipelines for nonclinical technologies, particularly those aimed at wellness or augmentation, should also be informed by rigorous “gold-standard” frameworks co-developed by regulators, scientists, and industry. While such technologies may be commercially driven or speculative in nature, transparency and relevance to the users’ benefits must remain central to their reporting standards. Here, collaboration between academic researchers and industry partners is essential, not only for generating robust empirical data but also for creating an evidence base that supports broader adoption. This is particularly critical in the wellness and consumer neurotechnology sectors, where devices often escape medical oversight and are seldom empirically validated.

However, the current landscape of neurotechnology regulation reflects a tension between the need for robust assurances regarding safety, efficacy, and data privacy and the urgency of making these technologies accessible and inclusive to clinicians, patients, and consumers (*205*). The lengthy and costly pathways for commercialization, regulatory approval, and ethical oversight often deter industry engagement, hindering the development of scalable and impactful neurotechnologies. A balance must be struck: enabling innovation while safeguarding users (*237*).

While existing programs like the Brain Research Through Advancing Innovative Neurotechnologies (BRAIN) (webBRAIN), Advanced Research + Invention Agency (ARIA) (Precision Neurotechnologies), and Horizon Europe (horizonWeb) broadly support neuroscientific research, dedicated initiatives focusing specifically on closed-loop neuromodulation are still needed. Recent efforts, such as the UK MHRA’s AIaMD Change Programme, the EU’s Artificial Intelligence Act and MDR, and the FDA’s Breakthrough Devices and Precertification programs, represent important steps toward regulating adaptive technologies. In June 2025, the UK introduced a major update to its medical device regulations, streamlining approval pathways and strengthening postmarket oversight, an important step toward aligning safety standards with the pace of neurotechnology innovation (*239*). Moreover, in April 2025, the UN Human Rights Council adopted a resolution endorsing a human rights approach to neurotechnologies (*239*). However, closed-loop neurotechnologies again introduce unique safety, accountability, and long-term use challenges. These features fall outside the scope of most existing frameworks. While technical standards such as IEC 60601-1-10 (covering physiologic adaptive controllers) and ISO 14971 (risk management for medical devices) provide important guidance, their adoption remains inconsistent, especially in nontraditional or early-stage applications. Addressing these challenges will require more targeted, internationally harmonized regulatory approaches that integrate standards for algorithmic transparency, continuous performance validation, and shared liability for both clinical and nonclinical applications.

Last, policies must anticipate societal consequences such as cognitive surveillance, inequitable access, and shifts in human autonomy (*237*). By aligning ethical responsibility with innovation pathways, we can support the safe and meaningful integration of open- and closed-loop neurotechnologies into clinical and everyday life.

### Call to action for stakeholders

The promise of neurotechnologies will only be realized through coordinated, cross-sectoral action. We believe:

1) Researchers and clinicians must adopt and refine robust, standardized protocols for both clinical and preclinical studies enabling reproducibility, meta-analysis, and translational insight. At the same time, fostering open-source development is essential to accelerate collaboration, promote transparency, and enable rapid adaptation across research and clinical settings.

2) Regulators and journal editors should coordinate efforts to enforce transparent reporting standards while preserving the flexibility needed to support innovation in emerging fields.

3) Industry partners, including those in the wellness and consumer neurotechnology sectors, must commit to sharing efficacy and safety data openly, especially for interventions that fall outside formal regulatory oversight. Wherever possible, companies should adopt open-source practices to promote transparency, accelerate innovation, and enable broader scientific scrutiny. Open-source development ensures that essential knowledge is not confined within the boundaries of individual commercial interests, whose priorities and stability may shift over time. This approach also facilitates interoperability, reproducibility, and collaboration across academic, clinical, and industrial domains.

4) Governments must invest in the manufacturing landscape at national and international levels to enable the translation of promising neurotechnologies from research to clinical implementation.

5) PPI should be embedded from the earliest stages of design, not as an afterthought, to ensure real-world relevance, equity, and public trust in neurotechnology deployment.

6) Funders must launch targeted initiatives to derisk the critical bottleneck between innovation and scalable manufacturing, a transition that currently stalls many promising neurotechnologies in preclinical stages. These initiatives should go beyond general neuroscience or medical device innovation, focusing specifically on the unique challenges of systems. Support should include long-term validation across diverse populations and sustained funding mechanisms for technologies deployed in decentralized or low-resource settings, ensuring equitable access and real-world applicability.

7) Investors must also recognize the unique timelines of neurotechnology and commit to “patient capital” models that extend beyond conventional 3- to 5-year return horizons. Traditional venture capital expectations are misaligned with the reality of clinical pilot trials, regulatory approval, and insurance adoption. Dedicated investment vehicles, hybrid funding models, and public-private partnerships are essential to sustain innovation through the long translational pathway. Without such patient capital, the field risks premature abandonment of promising technologies before they can reach patients.

8) Policy-makers must establish clear mandates for long-term oversight, data governance, and ethical accountability, especially in cases where responsibility becomes diffused after initial funding or deployment. Policies must enable innovation while ensuring equitable access, protecting autonomy, and preventing misuse, particularly in sensitive domains like cognitive modulation.

9) Last, mechanisms for postfunding responsibility and longitudinal oversight must be clarified. Long-term monitoring of systems often falls into institutional gray zones, risking both user safety and innovation stagnation. Sustainable frameworks are needed to ensure that responsibility for safety, efficacy, and equitable access persists beyond initial trials or grants.

We urge all stakeholders to move beyond siloed development toward a shared roadmap for the design, testing, and deployment of neuromodulation systems. In doing so, we can accelerate the creation of equitable, effective, and responsive technologies that adapt to real-time physiology, fundamentally reshaping patient care across a range of disorders. The time to act is now: Without coordinated effort, innovation risks outpacing both evidence and impact.

### Final notes

This roadmap reflects the collective insights of a specific group of ECRs who participated in a focused workshop at a particular point in time. While it captures a broad and interdisciplinary perspective, it does not represent an exhaustive or universally agreed-upon vision. The field of neurotechnology is evolving rapidly, and emerging discoveries, technologies, and societal needs may shift priorities in unforeseen ways. As such, this roadmap should be viewed as a living document, one that invites ongoing dialog, revision, and expansion as the field progresses.
